# Adeno‐associated virus (AAV)-based gene therapy for glioblastoma

**DOI:** 10.1186/s12935-021-01776-4

**Published:** 2021-01-26

**Authors:** Xin Xu, Wenli Chen, Wenjun Zhu, Jing Chen, Bin Ma, Jianxia Ding, Zaichuan Wang, Yifei Li, Yeming Wang, Xiaochun Zhang

**Affiliations:** 1 School of Medicine, Jiangsu University, Zhenjiang, 212013 Jiangsu China; 2 Department of Neurosurgery and Pituitary Tumor Center, The First Affiliated Hospital, Sun Yat-sen University, Guangzhou, China; 3 Department of Laboratory Medicine, The Second People’s Hospital of Lianyungang, Lianyungang, 222006 China; 4 Department of Hepatobiliary Surgery, The Second People’s Hospital of Lianyungang, Lianyungang, 222006 Jiangsu China; 5 School of Medicine, Yangzhou University, Yangzhou, 225600 China; 6 Department of Oncology, Yangzhou Traditional Chinese Medical Hospital, Yangzhou, 225600 Jiangsu China

**Keywords:** AAV, Glioblastoma, Gene therapy, Systemic injection, Local injection, Intracerebroventricular injection

## Abstract

Glioblastoma (GBM) is the most common and malignant Grade IV primary craniocerebral tumor caused by glial cell carcinogenesis with an extremely poor median survival of 12–18 months. The current standard treatments for GBM, including surgical resection followed by chemotherapy and radiotherapy, fail to substantially prolong survival outcomes. Adeno-associated virus (AAV)-mediated gene therapy has recently attracted considerable interest because of its relatively low cytotoxicity, poor immunogenicity, broad tissue tropism, and long-term stable transgene expression. Furthermore, a range of gene therapy trials using AAV as vehicles are being investigated to thwart deadly GBM in mice models. At present, AAV is delivered to the brain by local injection, intracerebroventricular (ICV) injection, or systematic injection to treat experimental GBM mice model. In this review, we summarized the experimental trials of AAV-based gene therapy as GBM treatment and compared the advantages and disadvantages of different AAV injection approaches. We systematically introduced the prospect of the systematic injection of AAV as an approach for AAV-based gene therapy for GBM.

## Introduction

Glioblastoma (GBM) is a tumor located in the central nervous system (CNS) that forms in the supportive tissue of the brain [1]. Human GBM is highly invasive and spreads rapidly to nearby healthy brain tissues before symptoms occur [2]. GBM has been reported to be the most lethal intracranial tumor because of its high resistance to conventional radiotherapy and chemotherapy [3]. Despite advances in surgery, the complex genetic heterogeneity and insidious infiltration of GBM cells result in almost inevitable recurrence with less than 5% 5-year survival rate [4]. Another major obstacle in GBM treatment is the blood–brain barrier (BBB), which limits the diffusion of most small-molecule therapeutic agents and all large molecules into the brain parenchyma and blocks the drug treatment of GBM [5]. Thus, developing effective therapeutic strategies that provide improved clinical therapeutic efficiency and increased survival rate among patients with GBM is urgently needed.

Gene therapy refers to the introduction of foreign genes into target cells to correct or compensate for diseases caused by defective or abnormal genes to achieve therapeutic purposes; this strategy is promising for many diseases, including cancer, neurodegenerative, and cardiovascular diseases [6, 7]. More than 2000 clinical trials of gene therapy have been conducted, and most of the vectors have been proven effective and safe [8]. Current studies indicate that approximately 64% of the clinical trials of gene therapy were conducted to treat cancer diseases, and the most common strategy is the delivery of tumor growth-inhibiting or tumor-killing genes [9]. RNA interference has been used in gene therapy to inhibit tumorigenesis and proliferation [10]. In addition, suicide gene [11], oncolytic virus [12], and immunomodulatory gene [13] have widely been applied in cancer gene therapy. The key to gene therapy is the use of safe and effective gene delivery vectors, such as viral and non-viral vectors. Fortunately, a variety of viral vectors including adenovirus [14], herpes simplex virus [15], and adeno-associated virus (AAV) [16] have been widely applied in the treatment of clinical and experimental cancer disease models. Among them, AAV, as an important viral vector, exerts a strong potential in the treatment of cancer diseases [17].

AAV vectors are promising in gene therapy for their stable, efficient, and non-cytotoxic gene delivery to transduce a great number of tissues of different mammalian species, including the CNS, and are one of the most commonly used viral vectors in gene therapy [18, 19]. Currently, AAV has been used as a vector for gene therapy in multiple clinical trials (more than 100) to target lung, liver, eye, brain, and muscle and has achieved great success in blindness and hemophilia diseases [20]. AAV1 vector-encoded lipoprotein lipase became the first gene therapy product (Glybera) approved to treat lipoprotein lipase deficiency by the European Union in 2012 [21]. Five years later, another AAV-mediated gene therapy drug (Luxturna) was subsequently approved for marketing in the U.S. [22]. Just last year, AAV9-based gene therapy (Zolgensma) has also been marketed to treat spinal muscular atrophy [23]. These development greatly inspired researchers to further explore the function of AAV as a gene therapy vector. AAV-mediated gene therapy strategies include gene replacement, gene silencing, and gene editing [17]. Recently, AAVs that deliver therapeutic agents have been utilized for the treatment of experimental GBM mice model and remarkably inhibited the growth of GBM cells and prolongs the survival rate of GBM mice [24]. Due to the presence of BBB, AAV that deliver therapeutic agents for the treatment of experimental GBM model are administered by intracranial local injection, which indeed relieves non-invasive experimental GBM in mice model [25, 26]. However, intracranial injection entails surgical risks and clinical costs and makes the scope of treatment relatively limited [27]. Human GBM cells are highly invasive and can migrate along blood vessels to areas of the brain away from the tumor bulk. This factor poses a big challenge for intracranial injection [28]. Researchers have also tried intracerebroventricular (ICV) injection to deliver AAV vectors to treat GBM. ICV injection can solve the relatively limited diffusion of AAV vectors in local injection to a certain extent and improves the therapeutic effect on invasive experimental GBM mouse model [29]. Instability and inevitable invasiveness are the drawbacks of ICV injection [30]. The development of BBB-crossing AAV make the systematic injection of AAV possible to treat GBM [31]. Systematic delivery, also called intravenous injection, can achieve widespread gene delivery and minimize invasive surgery; thus, this approach would be ideal for treating CNS diseases, including GBM [32, 33]. However, systematic injection in AAV-based GBM gene therapy also has some problems, including the low efficiency of AAV crossing the BBB, pre-existing AAV-neutralizing antibodies in the body, peripheral toxicity, and inability to target specific cells [34–37].

In this review, we systematically introduced the prospects of AAV-based gene therapy for GBM and compared the advantages and disadvantages of different AAV injection methods. Most importantly, we will focus on the feasibility of the systematic injection of AAV for the treatment of GBM and the challenge faced by systematic injection.

## AAV characteristics and its role in cancer gene therapy

### AAV structure and composition

AAV was accidentally found in the 1960s during a laboratory preparation of adenovirus and later found in human tissues [38]. AAV does not cause any human diseases, and its life cycle is connected with a helper virus (such as adenovirus and herpes simplex virus). AAV cannot replicate independently, and its replication and cytolytic functions can only be performed under the presence of helper viruses [39, 40]. AAV does not integrate with the host's genome and can stably express transgenes for a long period. In addition, AAV is widespread in many species, including human and non-human primates, and is highly infectious to a variety of tissue cells in vivo with non-pathogenic quality; thus, AAV has become the star vector for gene therapy [41, 42].

AAV is a single-stranded linear DNA-deficient virus with a genomic DNA of less than 5 kb, and its structure is icosahedral non-enveloped particle. AAV is composed of one single-stranded DNA with inverse terminal repeat (ITR) sequence and two open reading frames Rep and Cap at both ends. ITRs are symmetrical repeats that play important roles in the structure and function of AAV. The Rep gene comprises four overlapping genes Rep78, Rep68, Rep52, and Rep40 and can encode the Rep protein required for AAV replication, package, and genomic integration. Cap gene is composed of overlapping amino acid sequences and encodes the capsid protein, including VP1, VP2, and VP3 with a ratio of 1:1:10 (VP1:VP2:VP3). These three interact with each other to form a symmetrical icosahedron structure, which acts as a vehicle for gene delivery [43, 44].

### AAV-based cancer gene therapy

AAV-based gene therapy has been applied in a variety of preclinical and clinical trials to date and has shown a strong safety profile and trustworthy therapeutic effects [16]. In recent years, AAV has shown great value in the treatment of tumor diseases. Two clinical trials of AAV-based cancer gene therapy have been reported. One is the single injection of carcinoembryonic antigen (CEA)-specific cytotoxic T lymphocyte, which is activated by AAV2-CEA-transduced dendritic cells, to treat patients with advanced gastric cancer (ClinicalTrials.gov Identifier: NCT02496273), and the other is AAV2-hAQP1 applied in patients with squamous cell head and neck cancer (ClinicalTrials.gov Identifier: NCT02602249). In the treatment of cancer diseases, AAV can transduce a large number of cancer cells and cancer stromal cells and stably express cancer therapeutic genes (suicide gene, immunostimulatory gene, cytotoxic gene, small interference (siRNA) and anti-angiogenesis gene) to inhibit cancer formation and progression [45, 46]. The biggest problem with AAV-based cancer gene therapy is how to make AAV more specifically transduce to the cancer region [47]. Hence, a variety of rational designs of capsid have been engineered for cancer-specific transduction. Aminopeptidase N (CD13) is highly expressed in tumor tissues. Thus, Grifman et al. engineered AAV2 capsid by inserting an NGR peptide motif, which made AAV2 deliver therapeutic agents more efficiently and specifically to tumor cells [48]. Integrin is highly expressed on cancer vessels and cancer tissues and is used as an indicator of poor cancer prognosis. A study modified the AAV2 capsid by introducing a 4C-RGD peptide, which could efficiently combine αvβ3 and αvβ5 integrins. This modification promotes AAV2-mediated gene delivery to integrin-positive cancer cells in vitro and in vivo [49]. In addition, another study fused designed ankyrin repeat proteins to AAV2 capsid VP2 to target the cancer-associated receptor human epidermal growth factor receptor 2 (HER2)/neu. Her2-AAV selectively and highly transduces Her2-positive tumor cells and weakly transduces other cells, which greatly reduces its toxicity to other normal tissues [50]. AAV5 has also been engineered for cancer-specific transduction. Lee et al. engineered AAV5 with integrin-homing peptides, sialyl Lewis X and tenacin C, which are highly expressed in cancer cells [51]. Cheng et al. mutated tyrosine residues on AAV3 to phenylalanine, which increased the transduction capacity to hepatocellular carcinoma cells [52]. AAV capsid engineering promotes the effect of cancer cell-specific transduction to more effectively deliver therapeutic agents to the tumor site and greatly improve the treatment effect of AAV-based cancer therapy. The specific transduction of AAV is particularly important in AAV-based GBM gene therapy. How to make AAV specifically transduce to CNS regions and greatly reduce the peripheral toxicity of therapeutic genes especially in systematic injection approach are the key steps in AAV-based GBM gene therapy.

## AAV-based experimental trials on GBM mice model

**Fig. 1:**
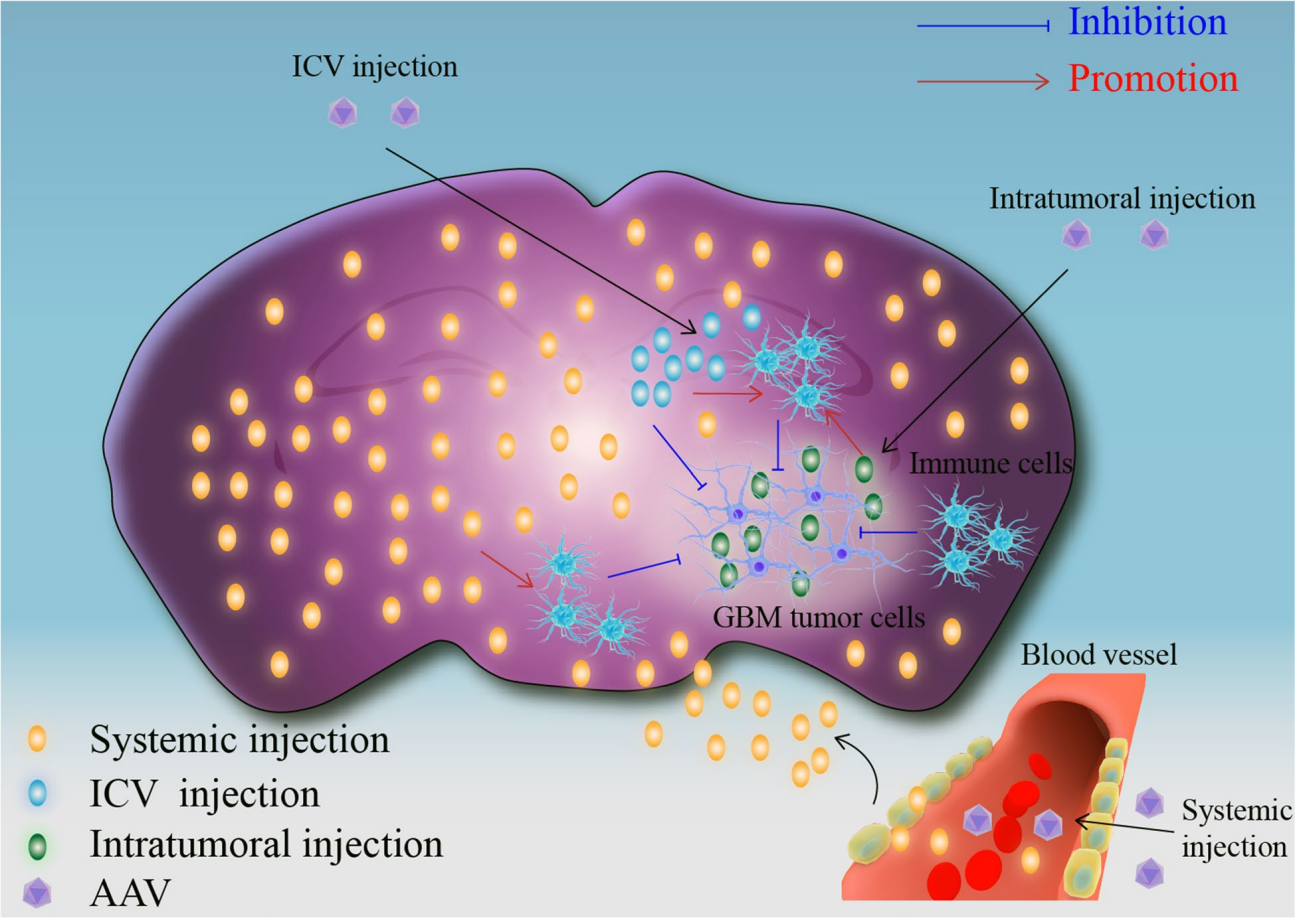
Different injection approaches of therapeutic AAV to treat GBM. Intratumoral injection is a common way to deliver therapeutic AAV to treat GBM in early years, but that has the limited transduction and surgical risk. ICV injection of therapeutic AAV can cause the widely transduction in the injected side, but it will lose the killing effect to the opposite side tumors. Systemic injection of therapeutic AAV will cause the widely transduced throughout the brain, and that can effectively inhibit invading GBM cells throughout the brain

AAV has been used to treat experimental GBM model for decades because of their stable and persistent expression of anti-tumor agents in transduced cells [53]. After the first discovery that AAV-encoded tumor suppressor genes could effectively inhibit the growth of GBM cell lines in vitro, AAV emerged as an effective delivery tool for the treatment of experimental GBM model [54]. Previously, AAV-based GBM therapy was administered by local injection because of the BBB, which blocks the path of AAV to the GBM [55]. Researchers have also tried the ICV route to deliver AAV directly into the cerebrospinal fluid to further penetrate into the brain parenchyma to treat experimental GBM mouse models and have achieved certain success [56]. The recent discovery of BBB-crossing AAV introduced a new approach, namely, the systematic injection of AAV, to fight GBM. Systematic injection seems a better treatment approach than local or ICV injection because of its non-invasiveness and broad transduction [57] (Fig. 1). AAV-mediated experimental gene therapy against GBM utilizes a variety of therapeutic strategies, such as tumor suppression and the use of anti-tumor genes, including anti-angiogenesis genes, cytotoxic or suicide genes, and immunostimulatory genes [58]. Next, we will systematically summarize the progress of AAV-based GBM research in several in vivo delivery routes and in vitro findings (Table 1).

**Table 1:** Different factors delivered by AAV to treat GBM

Factor	AAV serotype	Delivered approach	References
HSV-tk	–	Intratumoral injection	[62]
IFN-β	AAV2	Intratumoral injection	[63]
sVEGFR1/R2	–	Intratumoral injection	[25]
Anti-VEGF	AAVrh.10	Intratumoral injection	[64]
TFPI-2	–	Intratumoral injection	[65]
hTERTC27	–	Intratumoral injection	[66]
Decorin	AAV2	Intratumoral injection	[61]
TRAIL	AAVrh.8	Intratumoral injection	[67]
ADP	AAV2	Intratumoral injection	[55]
MiR-7	–	Intratumoral injection	[68]
IFN-β	–	ICVinjection	[29]
sTRAIL	AAV9	Systematic injection	[78]
IFN-β	AAV9	Systematic injection	[80]

### Anti-GBM effect of AAV in vitro

It is reported that the hypoxia-regulated AAV was first used to kill GBM cells in 2001 in vitro. They constructed a hypoxia-regulated AAV, which can encode the suicide gene *Bax* for the hypoxic GBM microenvironment. Their result showed that *Bax* was abundantly expressed under hypoxic condition after AAV transduction and promoted the death of GBM cells in vitro [54].Tumor necrosis factor-related apoptosis-inducing ligand(TRAIL), which induces tumor cell apoptosis but is less toxic to normal tissues, has been used in the treatment of various tumor diseases. Shawn et al. developed AAV-soluble TRAIL (sTRAIL), which could transduce GBM cells to promote the killing effect on GBM cells and increase pro-apoptotic protein level in GBM cells in vitro [59]. *PTEN* is the most common mutant tumor suppressor gene in various tumor diseases including GBM. Mutant *PTEN* rescued by gene editing can inhibit the proliferation of tumor cells. Thus, Victoria et al. developed AAV-mediated gene editing, which effectively modified the mutant *PTEN* gene in GBM cells and inhibited the proliferation and growth of GBM cells. The AAV-mediated killing of GBM cells in vitro indicates the feasibility of AAV-based GBM gene therapy [60].

### Anti-GBM effect of AAV through intratumoral injection

Intratumoral injection is the most generally preferred method for AAV to treat the experimental GBM mice model because of the presence of BBB. The local injection of AAV, which deliver therapeutic agents, has inhibited the growth of intracranial GBM and prolonged the survival rate of tumor-bearing mice to some extent [61]. AAV-based GBM therapy was first applied to treat experimental GBM mice model in 1996, Their study proved that a single intracranial injection of AAV-tk-IRES-IL-2 could effectively prohibit the progression of xenograft GBM. One year later their laboratory colleagues proved that the intracranial injection of AAV-tk and the intraperitoneal injection of gancyclovir could eliminate tumors in GBM mice [62]. However, despite its excellent result, the approach has serious hepatotoxicity, and its use has been stranded. Interferon-beta (IFN-β) has potent antitumor effects by inhibiting the growth and angiogenesis of cancer cells and promoting cancer cell apoptosis and immune stimulation. Since 2002, several researchers have tried to overcome the experimental GBM mice model by administering AAV-encoding IFN-β through local injection and have achieved certain effects [63]. Vascular endothelial growth factor (VEGF), as a pro-angiogenic factor, is remarkably upregulated in GBM tissue and promotes angiogenesis and growth of GBM tumors. Thus, AAV-delivered sVEGFR1/R2, a kind of VEGF-optimized soluble inhibitor, was used to treat experimental GBM model. Their result showed the powerful anti-GBM effect exerted by the local injection of AAV-sVEGFR1/R2 [25]. Furthermore, Some studies also found that bevacizumab, an anti-VEGF monoclonal antibody, delivered by AAVrh.10 could reduce the blood vessel density and volume of GBM tumor and increase survival rate [64]. Tissue factor pathway inhibitor-2 (TFPI-2) has a strong ability to inhibit tumor cell proliferation, migration, and angiogenesis. Niranjan et al. found that AAV-TFPI-2 could mediate the inhibition of GBM progression in vitro and in vivo [65]. It is reported that the overexpression of the C-terminal fragment of the human telomerase reverse transcriptase (hTERTC27) can prohibit the occurrence of malignant tumors including the experimental GBM model. Evidence has shown that intratumoral injection of AAV-hTERTC27 could inhibit the growth of xenograft GBM, amplify tumor necrosis and apoptosis, and reduce microvessel density in nude mice [66]. In addition, studies have shown that AAV2 intratumoral-delivered decorin, which exerted anti-tumor effect by affecting the epidermal growth factor receptor, transforming growth factor-beta, and p21, could inhibit GBM and prolong the survival of GBM mice [61]. Studies shown that AAV-secreted TRAIL (S-TRAIL) could promote the killing effect on GBM cells in vitro. Here, AAVrh.8-S-TRAIL accompanied with the administration of lanatoside C was proved to increase the overall survival of U87 bearing mice and that further confirm the anti-GBM role of S-TRAIL [67]. It is also clarified that AAV2-apoptin-derived peptide (ADP) promoted the apoptosis of GBM cells and prolonged the survival rate of orthotopic GBM bearing mice s[55]. Previous studies have shown that microRNAs could inhibit tumorigenesis. Here, studies have proved that AAV-miR-7 significantly reduced the tumor size, upregulated death receptor 5 to promote the tumor cell death and prolonged the survival in xenografts GBM mice model [68].

The intracranial injection of AAV circumvents the obstacles of BBB. Compared with the injection of pure therapeutic protein, AAV's persistent and stable expression of therapeutic agents can better inhibit the sustained development of GBM [69]. This was indeed the reason for the popularity of AAV-based GBM treatment approach in the early years. The above research also shows that the local injection of AAV delivering some traditional or GBM-specific anti-tumor genes is effective and, to some extent, alleviates the progress of experimental GBM mice model. Locally injected therapeutic AAV has been reported to infiltrate into the tumor area, but the AAV genome will be diluted because of the rapid growth and division of GBM tumor cells; this dilution will results in the reduced expression efficiency and affects the therapeutic effect [27]. In addition, studies have shown that the local injection of AAV with partial area transduction in the brain can only have a good effect on noninvasive, implanted GBM tumors, but human GBM is highly invasive. GBM cells can migrate along blood vessels away from the tumor core; thus, the local injection of AAV could hardly eliminate all the invasive distant GBM cells [70, 71]. Owing to the invasive nature of GBM cells, a globally spread gene delivery vehicle is badly needed to combat the diffused primary tumor or tumor recurrence. Studies have demonstrated proved that the injection of ssAAV2-ADP in the left hemisphere effectively prevents the growth of ipsilateral tumors but is not enough to prevent the growth of distal tumors in the contralateral hemisphere [55]. Furthermore, Matheus et al. showed that the intracranial injection of AAVrh8-sTRAIL indeed extends the survival rate of experimental GBM mice, but these mice also died of tumor spread within 100 days. Therefore, the local injection of AAV to treat human GBM is still very flawed and will not achieve the desired therapeutic effect. The researchers also demonstrated that the therapeutic gene must be widely expressed in the brain to fight against invasive GBM cells [67]. One way to achieve this goal is to systematically inject BBB-crossing AAV, which can perform extensive gene delivery in the brain.

### Anti-GBM effect of AAV through ICV injection

The ICV injection of therapeutic drugs is a common approach for the treatment of CNS diseases. In recent years, ICV injection is also widely used because it allows therapeutic drugs to reach most of the brain regions with the circulation of cerebrospinal fluid in the treatment of experimental GBM mice model [72]. Studies have shown that the ICV injection of AAV can overcome the disadvantages of local injection in GBM treatment. It is reported that intracranial fixed-point injection cannot completely eliminate distant infiltrating GBM cells because of the extensive infiltration and migration characteristics of GBM cells. They showed that the pre-injection of AAV vector encoding human IFN-β (AAV-IFN-β) through ICV injection can completely prevent tumor growth in an orthotopic model of GBM [29]. In addition, the survival rate of pre-established U87 intracranial tumor mice injected with AAV-IFN-β through ICV was substantially improved compared with injecting the control AAV vector through the same route. These data indicate that the ICV injection of AAV vectors that encode anti-tumor proteins is a promising method and deserves further study. Compared with local injection, the ICV injection of AAV can eliminate most of the distant GBM cells. Furthermore, local injection is highly dangerous when GBM is located in the critical structure of the brain, and ICV injection can well avoid this problem [73]. However, ICV injection also has defects. First, ICV injection is unstable. Second, ICV injection delivers AAV into the cerebrospinal fluid, which only circulates between the ventricles. The brain parenchyma area close to the ventricle may have good transduction, but the transduction efficiency for areas away from the brain ventricle may not be enough [56, 74]. Furthermore, other studies [29] demonstrated that AAV delivered by ICV injection also has chemotaxis in the brain, mostly transduces the hippocampus and corpus callosum, but rarely transduces other parts. From this point of view, AAV-based GBM gene therapy through ICV injection is also inadequate. Searching for a better delivery method that can make AAV transduce the entire CNS is a key step in AAV-based GBM gene therapy.

### Anti-GBM effect of AAV through systematic injection

The discovery of BBB-crossing AAV9 opened the door to the systematic injection of AAV for CNS diseases in 2009 [33]. BBB-crossing rAAVrh.8 and rAAVrh.10, which played a role in promoting the systematic injection of therapeutic AAV to treat CNS diseases, were discovered in 2014 [75]. To date, AAV9, AAVrh.8, AAVrh.10, AAVrh.39, and AAVrh.43 have been proved to have the ability to transduce glial and neurons after systemic injection [76]. AAV9 variants AAV-PHP.B and AAV-PHP.eB, which were developed by researchers through directed evolution approach, also have excellent CNS transduction ability in C57 mice [77, 78]. After the discovery of these BBB-crossing AAVs, researchers began to treat experimental GBM mice model with therapeutic AAV by systematic injection. It is the first time that the systematic injection of AAV was applied to treat GBM in 2016. Their result showed that systematic administration of AAV9-sTRAIL suppressed tumor growth and remarkably increased the survival of xenograft GBM mice [79]. Some studies also proved that systematic AAV9-IFN-β delivery could induce complete tumor regression in experimental GBM model in a dose-dependent manner. They also demonstrated that the systematic administration of AAV9-IFN-β is more efficient in multifocal GBM compared with local injection [80]. In recent years, the systematic injection of therapeutic AAV in GBM treatment has attracted considerable attention with the development of AAV9 variants AAV-PHP.B and AAV-PHP.eB, which have been proven to have a stronger ability to cross the BBB than AAV9.

The systematic injection of therapeutic AAV has extensive transduction characteristics and fundamental advantages over local injection or ICV injection; thus, this approach is an excellent way to treat GBM [80] (Table 2). A great number of studies have shown that systemically injected therapeutic AAV can transduce most regions of the CNS through the extensive vascular system and has a comprehensive containment effect on invasive, malignant GBM [81, 82]. It is reported that proved that the effect of the systematic injection of AAV9-IFN-βin treating multifocal GBM is better than that of local injection [80]. Moreover, ICV injection can only inhibit ipsilateral GBM tumors but not the tumors in the contralateral side because of its limitations in transduction [29]. These results clearly showed the advantages of the systematic injection of AAV in the treatment of GBM. Human GBM is highly invasive and can spread widely in the brain; thus, systemic injection is the best choice for AAV-based GBM gene therapy to eliminate GBM more thoroughly [83]. Despite its advantages compared with local injection and ICV injection, the systematic injection of AAV-based GBM gene therapy faces a variety of challenges that need to be resolved [84]. The first challenge is the efficiency of BBB crossing. BBB is the main obstacle that hinders the entry of therapeutic drugs into the CNS. How to overcome the BBB and transduce more efficiently into the CNS are the most critical steps in the systematic injection of AAV-based GBM gene therapy [85, 86]. Although some AAVs that can cross the BBB have been developed, more efficient AAV mutants still need to be studied. The second challenge is the non-specificity of AAV transduction. Systematic injection can widely distribute AAV in various parts of the body; hence, the expression of therapeutic gene in non-target cells away from the disease site is also very high and may result in ineffective treatment and high peripheral toxicity. The last challenge is the immune barrier [87]. Therapeutic AAV can be neutralized because of the large amount of AAV antibody in the human blood, which results in poor treatment effect or even no effect. Therefore, finding possible solutions to the challenges of systematic injection is the key in AAV-based GBM gene therapy.

**Table 2:** Comparison of different injection approaches of AAV-based GBM gene therapy

Injection approaches	Advantage	Disadvantage	References
Intratumoral injection	Overcomes the BBB obstacle	Transduces region limitation, Surgical risk	[70, 71]
ICVinjection	Good transduction for the brain parenchyma area close to the ventricle	Instability of ICV injection, Only the hemibrain has good transduction	[56, 74]
Systematic injection	Extensive transduction throughout the brain	Immune barrier, neutralizes antibodies in the blood	[76, 87]

## Conclusions and future prospects

GBM is a highly malignant intracranial tumor that is highly aggressive and heterogeneous. Surgical resection combined with radiotherapy and chemotherapy is the main method for the clinical treatment of GBM, but patient survival rate is still very low [88]. The development of gene therapy has been widely used in a great number of diseases. AAV has become a focus in gene therapy because of its stable, non-pathogenic, and long-term expression of therapeutic agents. AAV has been used for decades to deliver therapeutic agents to treat experimental GBM in mice model [89]. AAV-based GBM therapy is mainly administered by local injection in the early years because of the BBB [55]. Although this approach is damaging and can only achieve partial transduction in the CNS region, it plays a role in extending the survival rate of experimental GBM mice model. The discovery of BBB-crossing AAV9 in 2009 introduced the systematic injection of AAV to treat GBM. Systematic injection is noninvasive and has superior wide-spread transduction than local injection, especially for the treatment of highly aggressive tumors, such as GBM [33]. The systematic injection of therapeutic AAV has great advantages over local injection in the treatment of aggressive GBM [80] but also faces many challenges. Developing more efficient BBB-crossing AAV, performing AAV-specific CNS transduction, and reducing peripheral toxicity are the main challenges [87]. Researchers have used multiple genetic engineering techniques to make AAV capsid have the ability to cross the BBB and search for new BBB-crossing AAV serotypes [90]. Until now, a great number of BBB-crossing AAV mutants are being developed, including the AAV-PHP.B and AAV-PHP.eB, which can transduce the entire CNS region. Peripheral toxicity, especially liver toxicity, have been addressed through some countermeasures, such as inserting CNS-specific promoters or using microRNA to suppress peripheral transgene expression [91, 92], but cannot be completely eliminated. Thus, developing an AAV capable of CNS-specific tropism without infecting peripheral tissue is a direction worthy of further research. Compared with other viral vectors such as oncolytic viruses, AAV vectors have unique advantages. Although the oncolytic virus has a direct cytotoxic effect on GBM tumor cells, the AAV vector has the advantages of stability, high efficiency, and long-term continuous expression of therapeutic genes, which is more conducive to the durable inhibitory effect of therapeutic genes on GBM. Furthermore, AAV also has BBB-crossing ability, which poses the possibility of intravenous injection of gene therapy for GBM treatment, which is incomparable to other viral vectors. In conclusion, the systematic injection of AAV for the treatment of GBM is a promising direction, but some work needs to be studied further: developing more efficient BBB-crossing AAV, enhancing the CNS-specific transduction of AAV, and reducing peripheral toxicity.

## Data Availability

Not applicable.

## Abbreviations

GBM: Glioblastoma multiforme
AAV: Adeno-associated virus
ICV: Intracerebroventricular
CNS: Central nervous system
BBB: Blood–brain barrier
ITR: Inverted terminal repeat
VEGF: Vascular endothelial growth factor
TFPI-2: Tissue factor pathway inhibitor-2
hTERTC27: Human telomerase reverse transcriptase
ADP: Apoptin-derived peptide
miR: MicroRNA
DR5: Death receptor 5
